# Retraction Note: Targeted production of reactive oxygen species in mitochondria to overcome cancer drug resistance

**DOI:** 10.1038/s41467-026-74649-x

**Published:** 2026-06-26

**Authors:** Hai Wang, Zan Gao, Xuanyou Liu, Pranay Agarwal, Shuting Zhao, Daniel W. Conroy, Guang Ji, Jianhua Yu, Christopher P. Jaroniec, Zhenguo Liu, Xiongbin Lu, Xiaodong Li, Xiaoming He

**Affiliations:** 1 Department of Biomedical Engineering, The Ohio State University, Columbus, OH 43210 USA; 2 Comprehensive Cancer Center, The Ohio State University, Columbus, OH 43210 USA; 3 Davis Heart and Lung Research Institute, The Ohio State University, Columbus, OH 43210 USA; 4 Department of Mechanical and Aerospace Engineering, University of Virginia, Charlottesville, VA USA; 5 Division of Cardiovascular Medicine, Center for Precision Medicine, University of Missouri School of Medicine, Columbia, MO 65212 USA; 6 Department of Chemistry and Biochemistry, The Ohio State University, Columbus, OH 43210 USA; 7 Institute of Digestive Diseases, Longhua Hospital, Shanghai University of Traditional Chinese Medicine, Shanghai, 200032 China; 8 Division of Hematology, The Ohio State University, Columbus, OH 43210 USA; 9 Department of Medical and Molecular Genetics and Melvin and Bren Simon Cancer Center, Indiana University School of Medicine, Indianapolis, IN 46202 USA; 10 Fischell Department of Bioengineering, University of Maryland, College Park, MD 20742 USA

Retraction to: *Nature Communications* 10.1038/s41467-018-02915-8, published online 08 February 2018

This paper is being retracted after a joint research misconduct investigation conducted by Indiana University, The Ohio State University, and the University of Maryland, College Park found that Figure 7e and Supplementary Fig. 29 contain irregularities. The Editor has therefore lost confidence in this paper.

Jianhua Yu did not explicitly state whether or not they agree with this retraction.

Zan Gao, Daniel W. Conroy, Christopher P. Jaroniec, and Xiaodong Li agree with this retraction.

Hai Wang, Xuanyou Liu, Pranay Agarwal, Shuting Zhao, Guang Ji, Zhenguo Liu, Xiongbin Lu, and Xiaoming He did not respond to correspondence from the Publisher about this retraction.

